# Safe Use of Hyperbaric Oxygen Therapy in the Treatment of Diabetic Foot Ulcers: A Multidisciplinary Approach to Minimize Adverse Effects

**DOI:** 10.1155/2023/9154038

**Published:** 2023-07-25

**Authors:** Arman Vahabi, Merve Mert, Hüseyin Aytaç Erdem, Ilgın Yıldırım Şimşir, Meltem Işıkgöz Taşbakan, Anıl Murat Öztürk

**Affiliations:** ^1^Ege University School of Medicine, Department of Orthopedics and Traumatology, İzmir, Turkey; ^2^Ege University School of Medicine, Department of Infectious Diseases and Clinical Microbiology, İzmir, Turkey; ^3^Ege University School of Medicine, Department of Endocrinology, İzmir, Turkey

## Abstract

**Purpose:**

The purpose of this study is to evaluate the side effects associated with hyperbaric oxygen therapy and provide recommendations to prevent them in patients with diabetic foot ulcers.

**Introduction:**

The use of hyperbaric oxygen therapy in the treatment of diabetic foot ulcers remains a contentious issue, and minimizing side effects is critical. While the incidence of side effects related to hyperbaric oxygen (HBO_2_) therapy is low, it is essential to evaluate cases in a multifaceted and interdisciplinary manner to prevent adverse outcomes.

**Methods:**

A retrospective cohort study was conducted over the period of 2018–2020, involving a dataset of 100 patients. The primary objective of the study was to examine the frequency and types of side effects experienced by patients who underwent hyperbaric oxygen therapy (HBO) for diabetic foot ulcers (DFUs). In addition, we analyzed various wound characteristics, characteristics of hospitalizations, the surgical or medical interventions received by patients, and laboratory parameters including CRP levels, total blood count, culture results, HbA1c levels, duration of diabetes, treatment received for diabetes, and antibiotic therapy regimens.

**Results:**

The percentage of patients who experienced side effects was as low as 6%, and none of them were critical. The most common side effect was discomfort due to the confined space in the chamber.

**Conclusion:**

Appropriate patient selection, combined with a multidisciplinary approach to evaluate eligibility, is crucial to avoid adverse side effects. Patient education and early screening for side effects are also essential. Since various treatment protocols exist for HBO_2_ therapy, pooled data from different protocols may be misleading. Further studies focused on side effects with specific indications are necessary.

## 1. Introduction

Diabetic foot ulcers (DFUs) are chronic, non-healing wounds that occur due to accelerated factors that disrupt skin integrity on a favourable ground, created by macrovascular and microvascular changes caused by diabetes. Although risk factors, such as a history of amputation or foot ulcer, foot deformity, peripheral vascular disease, smoking, and others, as well as the pathogenesis are well known, DFU remains a serious and growing health problem [1, 2].

According to data from the International Diabetes Foundation, as of 2021, the prevalence of diabetes in the adult population is 10.5% [3]. The rate of increase in middle and low-income countries, including Turkey, is higher than that in high-income countries. The rate of diabetic foot ulcer development in patients with diabetes is around 10–15%. People with diabetes are 15 times more likely to undergo lower leg amputation than those without diabetes [4, 5].

In the management of diabetic patients, DFUs are one of the most challenging complications for both healthcare providers and patients. Given the multitude of sub-issues associated with the problem at hand, there is no universal solution. Chronic infections due to resistant microorganisms, the need for long-term wound care, and prolonged hospitalization, resulting in loss of labor and creating a psychological burden for both patients and caregivers, are just a few aspects of the problem. Such a problem with many components can only be solved with multiple treatment modalities and a multidisciplinary approach. In the debate of whether hyperbaric oxygen (HBO_2_) therapy has a place in this patient group or not, we believe that this method, which we have experienced, is safe and beneficial. If we can prevent the development of side effects, hyperbaric oxygen therapy has a place in the treatment of DFUs [6–8].

It is clear from the high rates of treatment failures and amputations that we have not yet achieved the desired outcomes in the treatment of diabetic foot wounds [9]. The search for optimal wound care and treatment modalities continues [10]. The use of hyperbaric oxygen therapy in the treatment of diabetic foot wounds remains controversial, despite its long history in this area. Although not all types of DFUs are listed as definitive indications in the guidelines of the Undersea and Hyperbaric Medical Society (UHMS), which is considered the primary reference for the use of hyperbaric oxygen, wounds with arterial insufficiency are still a major concern in the field. Given the pathophysiology of diabetic foot wounds, selected cases of DFU may fall under this category. As such, many centres that specialize in diabetic foot disease use hyperbaric oxygen therapy as an adjunctive treatment option in routine practice. In our clinical practice, we use hyperbaric oxygen therapy in selected cases of resistant diabetic foot wounds in addition to standard wound care treatments. The primary objective of our study was to evaluate the side effects of HBO_2_ therapy and provide recommendations to prevent them in DFU patients. It should be noted that studies on the side effects of HBO_2_ therapy with large datasets have included all patients with indications for HBO_2_ therapy, not just those with diabetic foot ulcers [11]. However, since diabetic foot ulcer patients generally have more comorbidities compared to other groups, there is a dearth of data on side effects that occurred specifically in this population.

## 2. Materials and Methods

The study was conducted retrospectively, with ethical approval obtained from the Ege University Ethics Committee. The researchers searched for patients who had been referred for hyperbaric oxygen therapy after a diabetic foot council decision between 2018 and 2020. Medical histories and laboratory results were obtained from consultation files and hospitalization files, and patients who had received hyperbaric oxygen therapy were included in the final analysis. Initial database search returned 108 patients. Patients with insufficient medical records and patients that referred to HBO with indications other than DFU were excluded. After excluding eight patients with insufficient medical records, data from 100 patients were included in the final analysis.

The researchers evaluated various wound characteristics, such as size, location, depth, leakage, and peri-wound skin characteristics, as well as the duration of the wound and changes in wound characteristics during the treatment process. We also looked at patients' hospitalizations, any additional surgical or medical interventions they received, and laboratory parameters, such as CRP levels, total blood count, culture results, HbA1c levels, duration of diabetes, treatment received for diabetes, and antibiotic therapy regimes.

To ensure that no data on side effects related to hyperbaric oxygen therapy were missed, the researchers conducted a combined search of digital hospital records, hardcopy records of councils, digital records of HBO_2_ centres, and national patient databases.

### 2.1. Council for Evaluation

In our tertiary-level university hospital, the Diabetic Foot Council meets once a week to organize the follow-up and treatment of patients with diabetic foot conditions. The council comprises faculty members from several departments, including orthopaedics, internal medicine, infectious diseases. All cases of diabetic foot wounds are followed up by consultants who confer with the council. In addition, a wound care nurse and a senior orthopaedic resident attend the council meetings. Outpatient follow-up forms are prepared as hard copies for documentation purposes, and these forms include patient history, clinical information, and schematic drawings used to evaluate wound healing progress so that the recovery of cases can be evaluated retrospectively.

Patient treatment is planned after considering several variables, such as the duration of the wound, its characteristics, blood sugar regulation, septic condition, patient adaptation capacity, the response from previous treatments, the presence of infection, cooperation with a caregiver, and accompanying medical conditions.

The decision of which patient is selected for hyperbaric oxygen therapy (HBO_2_) as an adjunctive therapy is made by the collective decision of council members. To avoid possible side effects, each consultant evaluates the patient in terms of their suitability for HBO_2_ therapy by conducting a deeper analysis of the patient's situation, vital capacity, and risk factors. The decision to provide the patient with the best possible option is made by considering absolute and relative contraindications for HBO_2_ therapy, limited capacity of the centre, additional cost of the treatment, and the possibility of delaying alternative optimal treatments. Our routine protocol for diabetic foot ulcers is with 2, 4 ATA pressure for 30 sessions of 30 minutes, but modifications can be made depending on the patients' characteristics.

The main contraindications of HBO_2_ therapy are untreated tension pneumothorax and recent use of doxorubicin, cisplatin, disulfiram, or mafenide acetate. Additionally, relative contraindications include decompensated congestive heart failure, uncontrolled lung disease, claustrophobia, recent ear surgery, upper respiratory tract infection, presence of a pacemaker, optic neuritis, otosclerosis, spherocytosis, and pregnancy. When evaluating a patient's suitability for HBO_2_ therapy, consultants are not limited to previously known diseases and can also ask personal questions about daily life activities that may predict heart and lung capacity.

**Figure 1:**
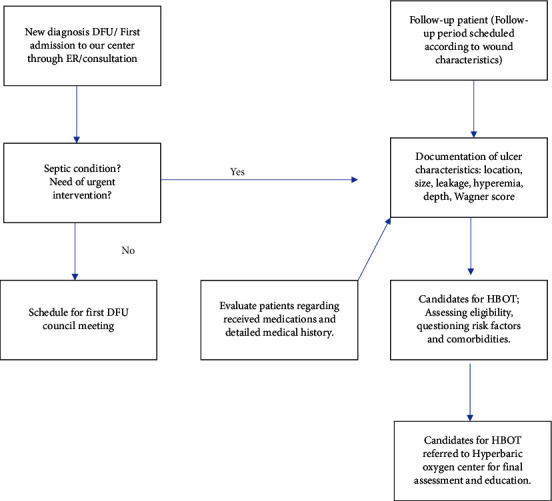
Patient management protocol.

At the end of the patient selection process, the patients directed to HBO_2_ therapy by the council are predominantly those with chronic diabetic foot wounds, wound site problems after surgery, diabetic foot wounds going through a plateau period in the healing process, and diabetic foot ulcers that do not receive optimal benefit from other wound care treatments. None of the surgical indications postponed HBO_2_ therapy trials. Patients in the grey zone for amputation level underwent an amputation level that is as distal as possible and were then referred to HBO_2_ therapy as an adjunctive treatment. Our approach is summarized in Figure 1.

## 3. Results

A total of 100 patients were included in the analysis, with a mean age of 63.1 (±10.8) years. The male to female ratio was 3 : 1, and hypertension (67%) and coronary artery disease (36%) were the most common comorbidities.

The mean duration of diabetic foot ulcers was 10.9 (±20.1) months, and 35% of patients had Wagner grade 4/5 ulcers (see Table 1). Out of the 100 patients, 61 received HBO_2_ therapy in the perioperative phase. Tissue or swab culture was obtained from 81 patients, with 58 out of 81 culture results being positive. The most common agents identified were *Pseudomonas aeruginosa* (19%) followed by *Escherichia coli* (15%) (see Table 2). Three culture samples resulted in *Candida albicans*. The mean number of HBO_2_ therapy sessions received was 27.1 (range: 1–60). The mean C-reactive protein (CRP) level was 76 mg/L (±67.98), and the mean leukocyte levels were 10,670/mm^3^ (±4,291). The mean HbA1c level before treatment was 9.7%. Hypertension and coronary artery disease (CAD) remained the most common comorbidities (see Table 3).

Side effects were retrospectively screened from medical records. Two authors (AV and MM) reviewed the medical records of all patients, searching for agreed-upon side effects such as middle ear barotrauma, transient myopia, claustrophobia, pulmonary oedema, exacerbation of congestive heart failure, and seizures.

Out of the 100 cases, six patients developed side effects. In 5 of these cases, treatment was discontinued due to side effects related to HBO_2_ therapy. Four of these cases were caused by fear or intolerance of the enclosed chamber, and one was due to ear discomfort in a patient with a history of middle ear surgery. Despite repetitive educational sessions, the patient was unable to cooperate on middle ear pressure equalization maneuvers (Table 4).

Four of the patients who experienced side effects were male, and all six were hospitalized in the infectious diseases ward. Additionally, 50% of all patients who received HBO_2_ were referred from that ward. During their treatment, all six patients underwent surgery for their wounds, with three of them having finger amputations, one with a Syme amputation, and two with transtibial amputations. Five of these patients had comorbidities, such as hypertension and coronary artery disease, in addition to their diabetes, while one did not have any other comorbidities.

One patient experienced ear pain during their second session and was diagnosed with middle ear barotrauma. The patient received treatment with a tube application and was able to complete their therapy sessions after the application.

## 4. Discussion

In terms of managing infections, we prefer to start empirical antibiotic therapy for local pathogens based on institutional preference after microbiological sampling of wounds that are suspected to be infected. We make this decision based on the Infectious Diseases Society of America (IDSA) recommendations for infected diabetic foot ulcers. Signs of infection include swelling/induration, erythema, tenderness, and warmth [12]. We revise the antibiotic treatment during follow-up appointments based on the culture results and the patient's clinical response. It is important to remember that appropriate antibiotic treatment for infected diabetic foot ulcers is crucial for successful multimodal treatment, and it has been shown to prevent high-level amputations and reduce mortality [10].

We believe that the individual differences in the pathogenesis of DFUs make it difficult to design comparative studies that accurately reflect the clinical benefits of HBO_2_ therapy. While DFUs are grouped together under one umbrella term, the underlying mechanisms and contributing factors can vary greatly from patient to patient. Although there are several wound classification systems for DFUs, these systems only provide limited information and do not account for patient-specific factors. Therefore, it is difficult to compare the effectiveness of different treatments across a large group of patients with DFUs.

It is possible that the conflicting results in the literature regarding the benefits of HBO_2_ therapy in DFU patients may be due to the individualized nature of the wounds and their pathogenesis. Even though patients may be classified under the broad category of DFU, each wound has its unique factors contributing to its development and progression. Therefore, well-designed studies comparing DFUs should focus on patients and wounds with similar characteristics, such as infection, size, depth, drainage, peri-wound skin, MRI findings, and culture results, to clarify the confusion in the literature. Multiple wound classification systems have been developed for DFUs; however, these can lead to complications when trying to pool patients for meta-analysis [13]. While arterial insufficiency is a common indication for HBO_2_ therapy in the treatment of chronic wounds, the pathogenesis of DFU suggests that most cases could benefit from adjunctive HBO_2_ therapy.

Overall, we believe that HBO_2_ therapy is a reasonable approach to treating most DFU cases. However, patient selection should prioritize safety and sustainability of resources.

It is worth noting that while Medicare covers the costs of HBO_2_ therapy in some cases, the availability and coverage of HBO_2_ therapy may vary depending on the country and healthcare system. While there have been some studies on the cost-effectiveness of HBO_2_ therapy in DFU patients, the data are limited, and further research is needed to draw global-scale economic conclusions [14, 15]. Moreover, the limited number of centres that offer HBO_2_ therapy means that only a select number of patients can receive this treatment. Therefore, selecting suitable patients is critical both for economic reasons and medical outcomes.

In general, patients tolerate HBO_2_ therapy well and the incidence of side effects that lead to discontinuation of treatment is significantly low. The side effects of HBO_2_ therapy are generally related to the toxic effects of oxygen and tissues that cannot adapt to pressure changes. Understanding Boyle's law can help to understand the mechanism of side effects. Boyle's law states that the pressure and volume of gas have an inverse relationship. Increasing pressure will result in decreasing volume. Changes in pressure mostly affect cavities in the body that are filled with air, such as the middle ear, pathologic lung cavities that are not connected to open air, and paranasal sinuses [16].

Pulmonary barotrauma, middle ear barotrauma, and paranasal barotrauma are all direct results of increased pressure. The most common pressure-related side effect is middle ear barotrauma, which can largely be prevented through patient education. Maneuvers such as the Valsalva maneuver, chewing, and swallowing can help balance the pressure in the middle ear by activating the Eustachian tubes. Prior to the session, patients should be informed and taught about these maneuvers to prevent serious pressure damage that could lead to swelling or even permanent hearing loss due to damage to the tympanic membrane [17]. Proper techniques for increasing the chamber pressure and otoscopic examination before treatment are also key factors in preventing this side effect.

Educating patients about the early signs of serious side effects and promptly detecting prodromal symptoms can help prevent further complications. Informing patients about potential early symptoms, such as shortness of breath, twitching, staring, visual and auditory hallucinations, nausea, dizziness, anxiety, and irritability, can help healthcare providers recognize side effects quickly and take immediate action.

## 5. Conclusion

Hyperbaric therapy is one of the safest therapies used in clinical practice. Most of the side effects are temporary and can be prevented with careful pretreatment evaluation. Common side effects include middle ear barotrauma, pulmonary complications, and ophthalmic complications, but these can be avoided through patient education and by carefully selecting patients with defined risk factors during pretreatment evaluation. A multidisciplinary approach and collaborative decision making are critical in all stages, including selecting appropriate patients for HBO_2_ therapy.

## Figures and Tables

**Table 1:** Distribution of wounds according to Wagner class.

	Frequency	Valid percent
Grade I	16	16.5
Grade II	22	22.7
Grade III	24	24.7
Grade IV	30	30.9
Grade V	5	5.2
Total	97	100.0

Total	100	

**Table 2:** Positive culture results.

Culture positive cases	*n* = 58
*Pseudomonas aeruginosa*	11
*Proteus mirabilis*	2
*Proteus vulgaris*	1
*Streptococcus agalactiae*	1
*Enterococcus faecium*	1
*Enterococcus faecalis*	7
*Klebsiella pneumoniae*	4
*Enterobacter cloacae*	2
*Acinetobacter baumannii*	4
*Staphylococcus aureus*	6
*Escherichia coli*	9
*Candida krusei*	1
*Candida albicans*	2
*Serratia marcescens*	2
*Morganella morganii*	1
*Corynebacterium striatum*	8
*Klebsiella oxytoca*	1
*Achromobacter xylosoxidans*	3
*Streptococcus dysgalactiae*	1
*Providencia rettgeri* ESBL(+)	1
*Proteus penneri*	1
*Enterobacter aerogenes*	1
*Citrobacter freundii*	1

**Table 3:** Comorbidities.

Comorbidities	*n* = 83
Hypertension	67
Chronic obstructive lung disease	6
Cerebrovascular disease	8
Chronic renal disease	16
Peripheral arterial disease	14
Congestive heart failure	8
Others	15

**Table 4:** Specific characteristics for patients that developed side effects.

Patient number	Age	Gender	Comorbidity	Side effect
1	55	Male	N/A	Intolerance of closed chamber
2	50	Male	DM, HT, KAH, COPD	Intolerance of closed chamber
3	69	Female	DM, HT, CAD	Intolerance of closed chamber
4	69	Male	DM, HT	Intolerance of closed chamber
5	73	Female	DM, HT	Middle ear discomfort
6	54	Male	DM, HT, CAD	Intolerance of closed chamber

## Data Availability

The dataset used to support the findings of this study is available from the corresponding author upon reasonable request.

## Abbreviations

MEB: Middle ear barotrauma
HBO_2_: Hyperbaric oxygen
DFU: Diabetic foot ulcer
QOL: Quality of life
CRP: C-reactive protein
CAD: Coronary artery disease.
